# Twitter-based crowdsourcing: What kind of measures can help to end the COVID-19 pandemic faster?

**DOI:** 10.3389/fmed.2022.961360

**Published:** 2022-09-16

**Authors:** Himel Mondal, Emil D. Parvanov, Rajeev K. Singla, Rehab A. Rayan, Faisal A. Nawaz, Valentin Ritschl, Fabian Eibensteiner, Chandragiri Siva Sai, Merisa Cenanovic, Hari Prasad Devkota, Mojca Hribersek, Ronita De, Elisabeth Klager, Maria Kletecka-Pulker, Sabine Völkl-Kernstock, Garba M. Khalid, Ronan Lordan, Mihnea-Alexandru Găman, Bairong Shen, Tanja Stamm, Harald Willschke, Atanas G. Atanasov

**Affiliations:** ^1^Saheed Laxman Nayak Medical College and Hospital, Koraput, Odisha, India; ^2^Ludwig Boltzmann Institute for Digital Health and Patient Safety, Medical University of Vienna, Vienna, Austria; ^3^Department of Translational Stem Cell Biology, Research Institute of the Medical University of Varna, Varna, Bulgaria; ^4^Institutes for Systems Genetics, Frontiers Science Center for Disease-Related Molecular Network, West China Hospital, Sichuan University, Chengdu, China; ^5^School of Pharmaceutical Sciences, Lovely Professional University, Phagwara, India; ^6^Department of Epidemiology, High Institute of Public Health, Alexandria University, Alexandria, Egypt; ^7^College of Medicine, Mohammed Bin Rashid University of Medicine and Health Sciences, Dubai, United Arab Emirates; ^8^Section for Outcomes Research, Center for Medical Statistics, Informatics, and Intelligent Systems, Medical University of Vienna, Vienna, Austria; ^9^Ludwig Boltzmann Institute for Arthritis and Rehabilitation, Vienna, Austria; ^10^Division of Pediatric Nephrology and Gastroenterology, Department of Pediatrics and Adolescent Medicine, Comprehensive Center for Pediatrics, Medical University of Vienna, Vienna, Austria; ^11^Amity Institute of Pharmacy, Amity University, Lucknow Campus, Lucknow, Uttar Pradesh, India; ^12^Independent Researcher, Sarajevo, Bosnia and Herzegovina; ^13^Graduate School of Pharmaceutical Sciences, Kumamoto University, Kumamoto, Japan; ^14^Headquarters for Admissions and Education, Kumamoto University, Kumamoto, Japan; ^15^ICMR-National Institute of Cholera and Enteric Diseases, Kolkata, West Bengal, India; ^16^Institute for Ethics and Law in Medicine, University of Vienna, Vienna, Austria; ^17^Department of Child and Adolescent Psychiatry, Medical University Vienna, Vienna, Austria; ^18^Pharmaceutical Engineering Group, School of Pharmacy, Queen's University, Belfast, United Kingdom; ^19^Institute for Translational Medicine and Therapeutics, Perelman School of Medicine, University of Pennsylvania, Philadelphia, PA, United States; ^20^Department of Systems Pharmacology and Translational Therapeutics, Perelman School of Medicine, University of Pennsylvania, Philadelphia, PA, United States; ^21^Faculty of Medicine, “Carol Davila” University of Medicine and Pharmacy, Bucharest, Romania; ^22^Department of Hematology, Center of Hematology and Bone Marrow Transplantation, Fundeni Clinical Institute, Bucharest, Romania; ^23^Department of Anaesthesia, Intensive Care Medicine and Pain Medicine, Medical University Vienna, Vienna, Austria; ^24^Institute of Genetics and Animal Biotechnology of the Polish Academy of Sciences, Jastrzẹbiec, Poland

**Keywords:** COVID-19, crowdsourcing, pandemic, public opinion, social media, Twitter

## Abstract

**Background:**

Crowdsourcing is a low-cost, adaptable, and innovative method to collect ideas from numerous contributors with diverse backgrounds. Crowdsourcing from social media like Twitter can be used for generating ideas in a noticeably brief time based on contributions from globally distributed users. The world has been challenged by the COVID-19 pandemic in the last several years. Measures to combat the pandemic continue to evolve worldwide, and ideas and opinions on optimal counteraction strategies are of high interest.

**Objective:**

This study aimed to validate the use of Twitter as a crowdsourcing platform in order to gain an understanding of public opinion on what measures can help to end the COVID-19 pandemic faster.

**Methods:**

This cross-sectional study was conducted during the period from December 22, 2021, to February 4, 2022. Tweets were posted by accounts operated by the authors, asking “How to faster end the COVID-19 pandemic?” and encouraging the viewers to comment on measures that they perceive would be effective to achieve this goal. The ideas from the users' comments were collected and categorized into two major themes – personal and institutional measures. In the final stage of the campaign, a Twitter poll was conducted to get additional comments and to estimate which of the two groups of measures were perceived to be important amongst Twitter users.

**Results:**

The crowdsourcing campaign generated seventeen suggested measures categorized into two major themes (personal and institutional) that received a total of 1,727 endorsements (supporting comments, retweets, and likes). The poll received a total of 325 votes with 58% of votes underscoring the importance of both personal and institutional measures, 20% favoring personal measures, 11% favoring institutional measures, and 11% of the votes given just out of curiosity to see the vote results.

**Conclusions:**

Twitter was utilized successfully for crowdsourcing ideas on strategies how to end the COVID-19 pandemic faster. The results indicate that the Twitter community highly values the significance of both personal responsibility and institutional measures to counteract the pandemic. This study validates the use of Twitter as a primary tool that could be used for crowdsourcing ideas with healthcare significance.

## Introduction

Crowdsourcing is the process of acquiring information or ideas from many individuals from diverse backgrounds for addressing a specific problem. The ideas from the “crowd” are often “sourced” through the internet (1). Members of the public may contribute ideas, review what other people are saying, and act on organizing committees. Thus, a large group of individuals engages in sharing diverse potential solutions for solving a given problem. Solutions proposed by a large group of individuals with diverse knowledge and background may provide alternative and highly creative solutions (2–4). Collating a wide array of diverse competing ideas can also represent a cost-efficient alternative to the traditional method of generating ideas. Novel ideas can be obtained from the public for the betterment of public health issues, thus empowering the initiative-taking participation of the public in elaborating superior public health solutions (5). Hence, crowdsourcing is being explored for generating ideas to solve public health challenges (6). Several previous studies have used crowdsourcing in global surveillance of diseases such as influenza, dengue, and malaria, diagnosis of diseases such as malaria and diabetic retinopathy, identifying the predictors of obesity or food choices in people, creation of awareness for the prevention of HIV transmission, measuring depression and other mental disorder *via* social media, among others (7). Crowdsourcing may be either paid or unpaid. Although paid ideas attract more contributors, there may be biased inputs. In unpaid crowdsourcing, the participants are motivated to participate without monetary compensation. Hence, the biases related to financial incentives are limited. Independently of the used crowdsourcing approach, relying on the wisdom of the public was shown to be far less expensive and less time-consuming than relying on selected specialists in all cases (8).

People have been utilizing social media to connect with family and friends, share information, express their thoughts on important topics, and engage in discussions during the COVID-19 pandemic. Several previous studies have evaluated the implications of using Twitter for disseminating COVID-19-related information (9, 10). As Twitter is an open social media platform, it gives the public instant access to a massive volume of diverse user-created content that may either contain credible health information or rumors, myths, and false information (11, 12). Most world leaders are using Twitter to transmit vital information on a myriad of issues including public health information to populations quickly (13). The World Health Organization (WHO) and other local health agencies are also sharing information on Twitter to make the public aware of appropriate health precautions and practices to adopt in order to end the pandemic. Hence, Twitter interactions may help build positive attitudes toward public health issues like acceptance of vaccines and promoting and maintaining a healthy and precautious lifestyle (14). In contrast, a piece of news from some questionable news websites may be shared by Twitter users to spread rumors or misinformation (15–17). For example, a research study showed that a conspiracy theory about COVID-19 has surfaced with a link to the installation of 5G network towers (18). Vaccine hesitancy is another crucial topic of interest in social media research (19). A recent study by Griffith *et al*. analyzed tweets to explore the major themes of vaccine hesitancy-related tweets from Canada. Such research may potentially help public health stakeholders in developing key policies for public health advocacy (20).

The crowdsourcing potential of social media like Twitter is not only restricted to the COVID-19 pandemic. Sowles and the collaborators had done the content analysis of vaping-related advertisements on Twitter (21). Alvaro and the team utilized the crowdsourcing Twitter annotations for the identification of adverse drug reactions for two kinds of drugs, selective serotonin reuptake inhibitors (SSRIs) and cognitive enhancers (22). Harris and the team studied the diabetes topics associated with engagement on a microblogging site, Twitter (23). Salazar-Carrillo and the team performed the traffic congestion analysis by performing data mining of traffic events from Twitter (24). Reuter and the team studied and analyzed public opinion for using social media like Twitter for clinical research-based activities (25). Koo and the team studied crowdsourcing on Twitter among urologists (26). Cutrell JB has shared his top 10 reasons for choosing Twitter-based crowdsourcing and #WhyID for infectious diseases. The top-most reason out of all was “You Join a Community That Loves What They Do and Passionately Advocates for All Patients No Matter What” (27).

In the last 2 years, the COVID-19 pandemic has spread across three waves in most countries (28). Despite all government efforts, there is still just limited success with the implementation of effective screening, tracing, and treatment modalities for COVID-19 at present. At this junction, the members of the public can play a vital role by contributing their ideas to formulate public health guidelines. In this context, the potential benefits of crowdsourcing applications become evident (29, 30). With a crowdsourcing approach, people from affected communities can submit their ideas, which would be further examined by experts for optimal implementation (31). However, in pandemic conditions, directly collecting data from a focused group meeting or a large group of individuals is difficult, time-consuming, and requires dedicated funds. In this situation, internet-based approaches may be of immense help through crowdsourcing using social media (32). To the best of our knowledge, no previous Twitter-based study was conducted to explore the potential of crowdsourcing for collecting ideas on how to end the COVID-19 pandemic faster.

This study aimed to validate the exclusive use of Twitter as a crowdsourcing tool on a timely and important topic, hypothesizing that a broad scope of valuable ideas could be collected based on the high number of users with diverse backgrounds that are active on this social media platform. Furthermore, this work serves to gain insights into public opinion on the kind of measures that can be adopted to help to end the COVID-19 pandemic faster.

## Materials and methods

**Figure 1:**
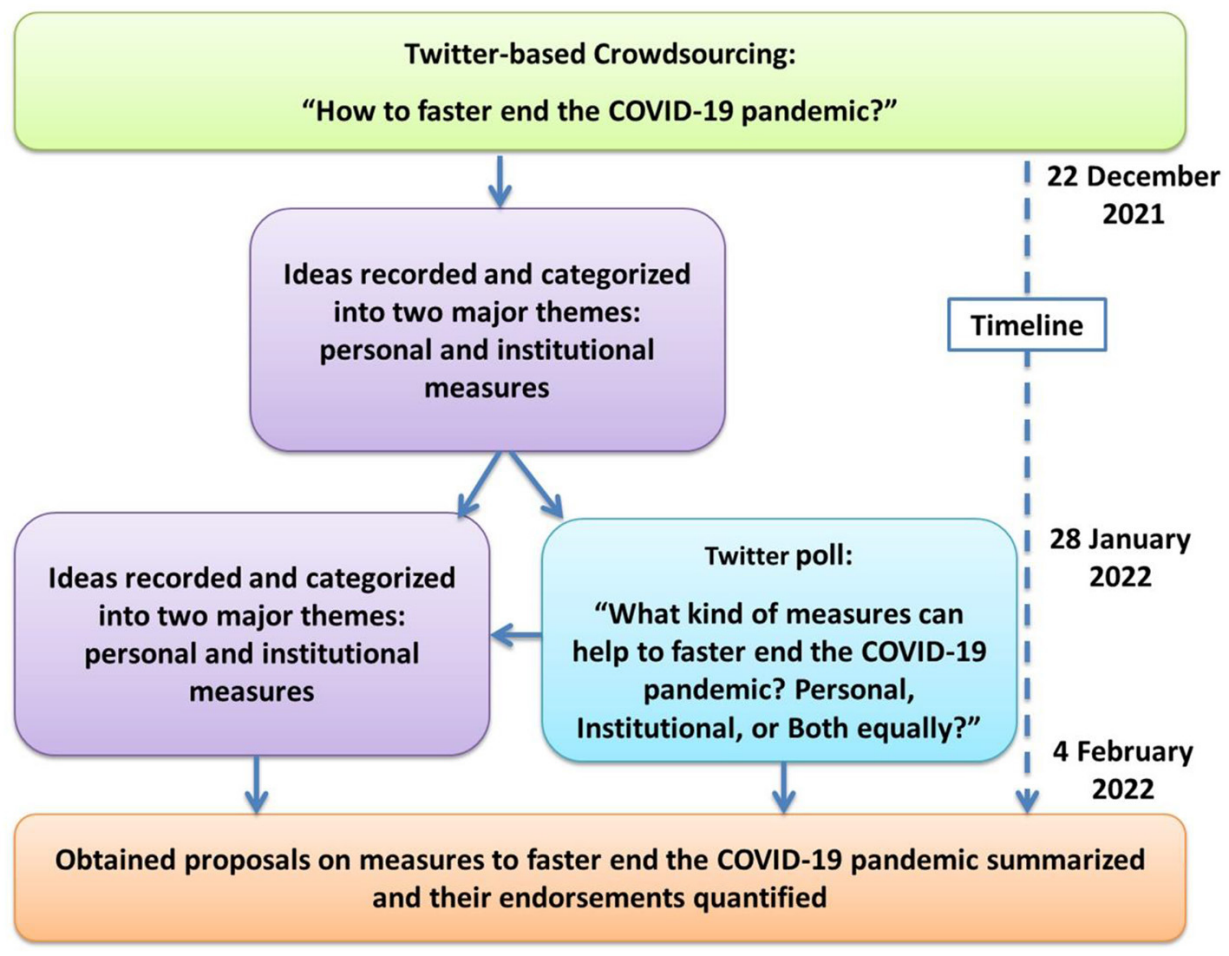
The applied study protocol, including the consequently conducted steps and the respective timeline.

This study involves crowdsourcing ideas on how to promptly end the COVID-19 pandemic from Twitter. The study was conducted during the period from December 22, 2021, to February 4, 2022. To gain further insight into the perception of Twitter users and additional feedback on proposed measures, a Twitter poll was conducted from January 28, 2022, to February 4, 2022 (during the last week of crowdsourcing). An overview of the study protocol is presented in Figure 1.

To explore the potential of Twitter to be exclusively applied as a tool to host crowdsourcing initiatives, authors used Twitter accounts operated by them (@DHPSP, @_atanas_, @ScienceCommuni2, @RajeevKSingla, @NawazFaisal_ai, @v_ritschl, @_Sivasai, @MojcaHri, @MerisaCenanovic, @_INPST, @Devkota_HP, @dronita_de) to share tweets asking the question “*How to faster end the COVID-19 pandemic?”* The tweets shared further incorporated short messages to encourage the viewers to comment on measures that they perceive to be most effective for ending the pandemic (e.g., “*Crowdsourcing for ideas: how to faster end the COVID-19 pandemic? Please comment below*”). The different ideas obtained in the form of comments or quoted tweets were recorded throughout the course of this campaign. The textual contents of the tweets were manually analyzed and the number of endorsements (supporting comments, retweets, and likes) associated with each idea was quantified by manual counting. As a first step, one of the authors (Atanas G. Atanasov) collected the engagements of Twitter users with the crowdsourcing campaign and grouped them in the preliminary list of categories. In a second step, the preliminary list was communicated by email to nine other authors and subjected to further refining based on received feedback. The latter process continued until all ten authors achieved unanimous consensus on all collected measure categories. The suggested measures were further grouped into two general themes-personal measures, and institution-mediated measures. For example, personal responsibility-related measures include compliance with recommended measures or making personal lifestyle modifications to minimize the chance of contracting COVID-19 and strengthening general health. On the other hand, examples of institutional actions include support for the development of a portfolio of vaccines and pharmaceuticals, securing universal healthcare accessibility, and provision of free tests and consumables (33).

**Figure 2:**
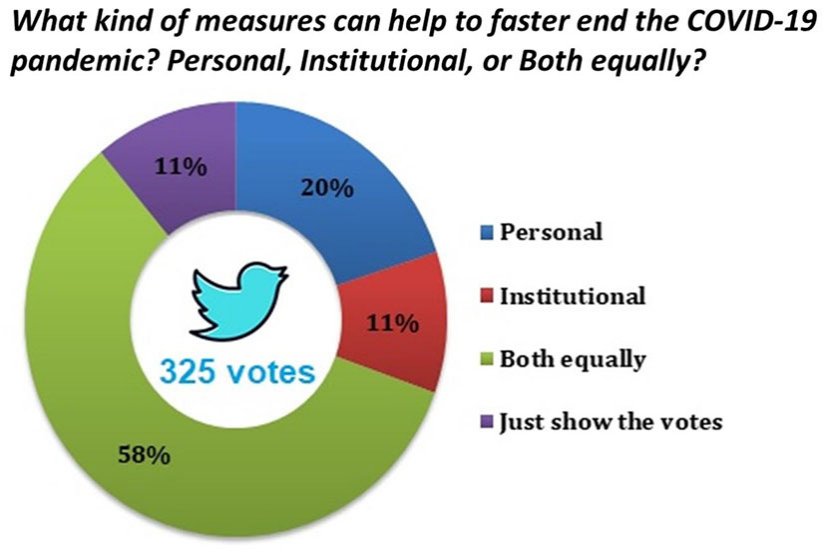
Twitter poll result reflecting the opinions of users on the perceived importance of personal vs. institutional measures to quickly end the COVID-19 pandemic. The data were obtained from the Twitter analytics feature of the DHPSP Twitter handle on February 12, 2022 (Chi-square test [compared the number of votes in each response option with an expected equal number of responses in each option; statistical significance indicates that the votes in four response options did not occur by chance]: χ^2^ [3] = 201.5, *P* < 0.0001).

To gain further input from Twitter users on which of these two groups of measures are considered more important, as well as to get additional feedback and visibility for the crowdsourcing initiative, a dedicated poll was developed and pinned on the Twitter account (@DHPSP) of the Digital Health and Patient Safety Platform (DHPSP; an open innovation platform aiming to stimulate the application of digital technologies for the promotion of public health) (34). The poll was open for voting for seven days (during the last week, in which the crowdsourcing was open for collecting ideas; from 28 January to 4 February 2022). The poll asked the following question: “*What kind of measures can help to faster end the COVID-19 pandemic? Personal, Institutional, or Both equally?”* (Figure 2). Twitter users were further encouraged to both vote and leave further comments on the measures they support. The four response options were: “Personal;” “Institutional;” “Both equally;” and “Just show the votes.” In relation to this poll design, it should be noted that Twitter just allows up to four answers per poll, with a limit of twenty-five characters (including spaces) for a response option (35). The vote counts were recorded after the poll was closed (on February 12, 2022) and the received comments were recorded and included in this crowdsourcing study.

No ethical approval was required for this study, as it does not fall within the scope of the Austrian Medical Ethics Act (analyses of publically available, de-identified data do not require ethical approval in Austria, and authors' institutions do not have specific policies which would overrule the latter regulation). All tweets with crowdsourcing ideas are publicly available on the web (Twitter). The votes of the conducted poll are anonymous (an intrinsic feature of Twitter polls), and anonymization (removal of Twitter user data) was applied to all collected data before performing the analysis presented in this work.

## Results

Ideas or measures generated from the conducted Twitter crowdsourcing are listed in Table 1 (including quantification of received endorsements), and a more explicit description of the crowdsourced recommendations is provided in the following paragraphs.

**Table 1:** An overview of the obtained proposals for measures that might be helpful to quickly end the COVID-19 pandemic and their respective endorsements.

**Measures**	Endorsements*(including original comments)
**Personal**	
**Trust in experts and compliance with recommended measures** [trust in expert opinion (e.g., healthcare professionals), ignoring information from unreliable sources, adherence to the measures recommended by experts]	167 (13)
**Lifestyle modifications** (avoidance of crowded areas, digital payments, alternative greeting methods such as “Namaste,” intake of nutritios foods, physical activity, sunlight exposure)	101 (7)
**Nutritional supplements and traditional medicine remedies** (nutritional supplements to strengthen immunity, traditional herbal medicine for economically deprived nations)	83 (11)
**Care for others and social responsibility** (protecting of others, community contact tracing, listing of exposure locations)	80 (7)
**Mental adjustments** (building of appropriate mindset, encouragement of mental attitudes oriented toward stress reduction)	40 (3)
**Institutional**
**Healthcare accessibility and equity** (securing of access to vaccines and tests, disease prevention education, coordinated health policies, global efforts toward healthcare equity and transparency in information)	191 (13)
**Portfolio of vaccines and optimized immunization strategies** (portfolio of diverse vaccines, combined vaccination strategies, vaccines with different mechanisms of action, application of COVID-19 vaccination calendars, breast milk sharing banks)	189 (13)
**Infrastructure changes and associated strategic funding** (infrastructure changes such as implementation of better quality air filter installations and better ventilation in public buildings, warm water availability in washrooms, strategic funding for viral sequencing, institutional psychological care efforts)	157 (11)
**Public dialog, science-based education policies, and presentation of more transparent data** (more pronounced public dialog, science-based education and communication efforts, promotion of policies that are based on science, availability of more transparent and unbiased data analysis)	152 (13)
**COVID-zero approach and a stronger focus on containment** (strict quarantine measures, massive testing, more efforts toward isolation of positive cases, restrictions toward traveling or gatherings, regional or sesonal lockdowns, more efforts toward contact tracing)	140 (12)
**Free or affordable tests and N95 masks** (free or affordable COVID-19 test, including at-home tests, free N95 respirators or similar quality masks, attractively designed masks)	138 (10)
**Limiting of commercialization** (limiting the commercialization of technology and materials related to COVID-19, emergency access to relevant technology and materials, facilitating cheaper vaccine manufacturing in developing countries, independent overseeing of pharmaceutical programs aimed at the development of COVID-19 therapies and vaccines)	65 (5)
**Focus on disinfectants and agents that prevent infection** (promotion of infection-preventative agents such as nasal sprays or mouthwashes, application of diverse modern technologies for disinfection)	61 (5)
**More focus on the rapid development of pharmaceuticals** (focus on rapid development pharmaceuticals, therapeutic antibodies, expedited approvals by regulatory agencies such as FDA, EUA)	46 (5)
**Penalties and rewards associated with adherence to recommended measures** (personal incentives (including financial) for vaccination and compliance with recommended preventive measures, penaltization for non-complience with recommended measures)	42 (3)
**Focus on early disease detection** (early disease detection, next generation of viral sensors or detectors)	42 (4)
**Focus on early treatment** (alternative early treatments)	33 (5)

*Endorsements were calculated as the combined number of supporting comments, retweets, and likes on Twitter; the number of original comments suggesting the respective measure is indicated in parentheses.

### Personal measures recommended by twitter users

#### Trust in experts and compliance with recommended measures

To faster end the COVID-19 pandemic, Twitter users perceive and endorse that people should have more trust in expert opinion (e.g., healthcare professionals) and ignore rumors and false information about vaccination against COVID-19. When someone is exposed to COVID-19 patients or tests positive, they should adhere to the recommended measures indicated by specialists, such as masking, distancing, immunization, hand sanitizing, testing, and isolation/quarantine.

#### Lifestyle modifications

To minimize the chance of contracting the disease, lifestyle modification measures such as avoidance of crowded areas, more digital payments instead of using cash, the practice of alternative greeting methods (“Namaste”) instead of handshakes, an attempt to minimize physical contact with others, and preference for dining at home or in open spaces. Lifestyle modification measures such as intake of nutritional foods to improve immunity, sufficient physical activity, optimum sleep, and adequate exposure to sunlight can be adopted for strengthening general health.

#### Nutritional supplements and traditional medicine remedies

To prevent and combat COVID-19, nutritional supplements like vitamins and minerals may strengthen the immune system. In addition, there is a potential value of traditional medicine [e.g., traditionally used herbal remedies (36)] in world regions where it is often difficult to get access to modern healthcare, including COVID-19 vaccines (e.g., many regions in Africa).

#### Care for others and social responsibility

Awareness should be increased of an individual's responsibility and accountability regarding the health of other people. Citizens should be made aware that “freedom” is not equal to “selfishness.” A personal priority should be not just how to protect oneself, but also how to protect others. Another measure related to taking social responsibility that was proposed was the involvement with community contact tracing for early detection of exposures, which can be done *via* social media groups when state government health departments cannot keep up with outbreak contact tracing and listing of exposure locations. It was also suggested that more people may comply with required measures if they are properly informed and left with “free choice” (concerning the notion that trying to force specific measures often has the opposite effect and may induce more resistance than compliance).

#### Mental adjustments

It was pointed out that immunity is influenced by the mind and the right mindset may positively influence it. Along this line, it is well-known that stress-induced hormones (including glucocorticoids, which are among the most potent endogenous immunosuppressive agents) have detrimental effects on immune functions (37). Thus, Twitter users recommended the encouragement of positive mental attitudes oriented toward stress reduction (not to be excessively scared and panic, to focus on fulfilling activities such as work and on activities associated with positive emotions).

### Institutional measures recommended by the twitter users

#### Healthcare accessibility and equity

A greater emphasis on the equity and universal accessibility to healthcare internationally, including access to vaccines, tests, and prevention education, was recommended. The same focus should be extended to rural areas within developed countries. It was further explicated that a worldwide consensus and global equity efforts can be beneficial on issues such as (a) vaccines and treatment; (b) coordinated health policies; and (c) transparency in information and commitment to sharing that information with the general population. Moreover, such efforts should include support for low-income countries in controlling the pandemic.

#### Portfolio of vaccines and optimized immunization strategies

Specifics related to the development, distribution, and implementation of vaccination programs are of high importance. It was suggested that a portfolio of vaccines that would target more than one viral protein should be available. The potential of combined vaccination strategies (e.g., combining mRNA vaccine with vector vaccine in one shot or offering a combined vaccine simultaneously targeting influenza and COVID-19) was also outlined. Furthermore, the promise of polyvalent inactivated vaccines and the benefit of a vaccination strategy that would prioritize vaccination of vulnerable population groups should be emphasized. The development of aerosolized vaccines that can be used for large-scale vaccination campaigns in specific settings (with aerosolized inoculation that closely matches natural exposures in public spaces to the aerosolized virus from people breathing nearby indoors) was also suggested. An emphasis should be made on the development of a COVID-19 vaccination calendar in each country. Economical vaccines should be available to all corners of the world. Further approaches that were proposed were the development of plant-based vaccines and the creation of breast milk-sharing banks to spread inoculated milk to more children that are too young to be vaccinated.

#### Infrastructure changes and associated strategic funding

The potential of infrastructure modification was also endorsed as an important institutional measure. These measures include the incorporation of higher standards in building codes such as quality air filter installations in public buildings, ultraviolet (UV) air sanitizers in heating, ventilation, and air conditioning (HVAC) systems along with other technologies for disinfection of indoor spaces such as cold plasma. There is a need for better ventilation in public spaces, and ensuring the availability of warm water for washing in public buildings (including bathrooms, if not available yet). Furthermore, strategic funding should be allocated to infrastructure and resources for laboratories for ease of viral sequencing to survey novel variants. The importance of psychological care efforts at the institutional level was also pointed out. For example, allowing hospital visitors with proper personal protective equipment to meet their hospitalized relatives to promote the psychological benefits to both the patients and their relatives, and integrating specialized trauma and grief-orientated mental health services for healthcare workers.

#### Public dialog, science-based education policies, and presentation of more transparent data

It was recommended that there should be a more pronounced public dialog involving a better explanation of recommended measures and guidance on prevention strategies. Also, the importance of science-based education and communication efforts to provide reliable information on scientifically proven approaches for prevention and therapy is essential. Additionally, the need to understand and eliminate communication gaps between anti-vaxxers and the scientific community is crucial. The benefits of policies that are based on science and not on political motivation were also endorsed. The use of broadcasting science-based educational video clips was proposed. There should be more transparent and unbiased data analysis that will bring more clarity to the public health situation. Remote clinical trials were proposed as a powerful tool that can yield valuable big data.

#### COVID-zero approach and a stronger focus on containment

The COVID-zero approach may be a reasonable choice to counteract the spread of COVID-19. COVID-zero strategy was adopted by China, and it is characterized by strict quarantine measures, massive testing, isolation of positive cases, travel restrictions, and regional lockdowns (38). It was reasoned that the adoption of measures resembling the epidemic policy of East Asian countries would be of benefit. Also, better protocols for the containment of the spread of the disease, including strategic testing, entry restrictions and quarantines, and contact tracing (through smartphone applications or other approaches) would help to end the pandemic. Another measure that was suggested was the introduction of a prophylactic lockdown in winter (similar to winter breaks in schools). It was underlined that measures counteracting the crowd gathering are of particular importance. It is also important to continue focusing on shielding vulnerable populations by establishing clinical isolation settings between different patient groups inside hospitals.

#### Free or affordable tests and N95 masks

The tests for COVID-19 should be free, or at least they should be accessible at an affordable rate. It would be beneficial if there was facilitation for the conductance of at-home tests for SARS-CoV-2 for everybody, or with priority to specific sections of society–for example, families that have children visiting a school. Also recommended was the distribution of free N95 respirators or similar quality masks for everybody, or with priority to specific sections of the society (e.g., to the healthcare workers or to older people with chronic diseases). If the masks are painted with attractive designs, for example, with the national flag logo (or cartoon characters on masks designed for kids), it might make the mask more popular and more adopted. It was also emphasized that masks with higher filter capacity (e.g., N95 or KN95 masks) are providing better protection than masks offering lower filter capacity (e.g., cotton or surgical masks), and therefore the use of the former group of masks should be encouraged.

#### Limitation of commercialization

It was recommended that appropriate efforts should be made by the authorities to prevent or limit the proprietary or exclusive rights-based commercialization of anything related to COVID-19. Furthermore, emergency access to relevant technology and materials regardless of intellectual property copyright should be available for the public interests (e.g., releasing patents related to vaccines would be of remarkably high benefit). Also, the benefit of facilitating cheaper vaccine manufacturing in developing countries without impediments was suggested. Independent overseeing of pharmaceutical programs aimed at the development of COVID-19 therapies and vaccines was also suggested.

#### Focus on disinfectants and agents that prevent infection

The development and application of infection-preventative agents such as nasal sprays or mouthwashes, as well as the potential role of modern technologies for disinfection, was deemed important.

#### More focus on the rapid development of pharmaceuticals

It was suggested that promoting a stronger industry focus on rapid medication development would be of a strong benefit. This also includes faster progress with the development of therapeutic antibodies. Furthermore, there should be expedited approvals of therapeutic preparations by regulatory agencies (e.g., FDA, EUA).

#### Rewards and penalties associated with adherence to recommended measures

The introduction of reward systems for those who adhere to recommended measures to combat COVID-19 would be a positive reinforcement. For example, personal financial incentives for each person who received a vaccination shot may help attract people to get vaccinated. In addition, those who do not comply with recommended measures like vaccines or masks may be penaltized.

#### Focus on early disease detection

Better protocols for early detection of the disease, including the development of new detection technologies such as the next generation of viral sensors or detectors for air quality sampling for crowded areas, may help in the early detection of the disease outbreaks (39).

#### Focus on early treatment

The potential importance of the timely application of alternative early treatments that should be applied before the disease worsens was also highlighted.

## Discussion

Intending to substantiate the use of Twitter as a social media for crowdsourcing, in this work we successfully generated a broad range of ideas from a large international pool of users. The Twitter users who participated in this crowdsourcing campaign did not receive any monetary compensation. The crowdsourcing campaign was promoted through multiple Twitter handles operated by the authors, some of which with high numbers of followers (e.g., @DHPSP: 1986 followers, @_atanas_: 118,111 followers, @rajeevksingla: 1,857 followers, @ScienceCommuni2: 1,073 followers) that is far above the median followers of an average Twitter account (i.e., around two hundred followers is the median) (40). This may be one of the reasons why numerous Twitter users were able to see the tweets about the crowdsourcing being conducted, which allowed various individuals to share their ideas.

Twitter was previously utilized in diverse ways for surveying the opinions of users in the context of the COVID-19 pandemic. Monitoring of Twitter discussions and Twitter polls, for example, was previously applied to find the level of acceptance and hesitance to take vaccines among users (13, 32, 41, 42). Adding to the literature of healthcare research based on Twitter in the context of COVID-19, the current study explores the potential of Twitter for crowdsourcing ideas from Twitter users by directly tweeting a specific question. Noteworthy, this crowdsourcing was done at the stage of the pandemic when most of the countries in the world had already witnessed three waves of the pandemic. Hence, the knowledge base of people, as well as Twitter users, has been enriched through previous exposure to various sources and considerations. Thus, Twitter users could tap into their accumulated knowledge, experiences, and expectations to generate multiple ideas and share them in the frame of the conducted Twitter crowdsourcing.

As already briefly mentioned, along with the here-applied crowdsourcing approach of directly tweeting a question, the application of a Twitter poll is another method to survey user opinions (43). In this study, in the last week of crowdsourcing, a poll was posted, and votes were collected along with additional crowdsourcing ideas. Thus, users who might have missed the tweets with the previously posted crowdsourcing question may land on the poll, cast a vote, and share additional ideas.

There are several advantages and disadvantages of Twitter as a medium for crowdsourcing healthcare-related ideas. A major advantage is a speed of data collection from all potential respondents from any corner of the world. Experts from various backgrounds can contribute to crowdsourcing (44). Volunteers can in this way contribute ideas that can have potential impacts on the betterment of healthcare. Although market research crowdsourcing may conventionally contain monetary compensation, as illustrated well by our work, healthcare-directed crowdsourcing may not require alluring users with gifts or financial incentives. Another advantage of the utilized approach is the simplicity of both posting tweets with specific questions on the part of the campaigner, as well as the ease of response by Twitter users. Since smartphones and the internet have already reached an overly broad worldwide use, the applied approach also enables the collection of ideas at a broad scale internationally.

There are certain disadvantages to Twitter's use as a medium for crowdsourcing health-related information. There are limited demographic data mentioned in the biographies of specific Twitter users (on some occasions location, gender or profession might be indicated; age is mentioned even more rarely), and the authenticity of the declared user identity cannot be verified for the majority of the respondents' accounts. As a result of such anonymity, some users may liberally communicate dubious ideas and the information received might not be reliable in some cases (45). Therefore, to counteract such tendencies, during crowdsourcing moderation by experts with broad subject-specific knowledge is necessitated for filtering promising ideas (although such moderation is needed for every kind of crowdsourcing, it must be acknowledged that anonymity offered by social media platforms results in a higher degree of communication of unreliable information and dubious ideas). Another disadvantage of using Twitter for public opinion surveys is that the users that will have initial exposure to asked questions are limited to the members of the pre-established networks of the researchers conducting the surveys. With further broad re-tweeting of such shared questions, such initial effects are diminishing with time since each following retweet increases the visibility of the initially shared tweet among the users of the retweeting accounts. Nevertheless, such initial effects must be taken into consideration. One way to diminish such initial effects in future studies could be to use the paid Twitter Advertising service to broadly increase the visibility of Twitter crowdsourcing campaigns. Such an approach might also counter visibility differences that might arise from Twitter algorithms favoring the display of the specific type of tweets (but not other types) in the feeds of single users.

While conducting digital public health surveys and crowdsourcing campaigns, it is important to consider that the representation of users of specific digital media (in this case Twitter), does not fully mirror the representation of diverse societal groups (e.g., in respect of age, sex, ethnicity, and disability status) (46–48). Along this line, obtained results should be interpreted with caution and awareness that not all social groups are equally represented on Twitter. Moreover, future Twitter-based crowdsourcing campaigns might address such disparities by making a special effort to reach groups that are known to be less represented among Twitter users.

Among the five personal measures that were yielded from crowdsourcing, the highest endorsement was received for keeping trust in expert opinion available at that point of time and being compliant with the recommended measures by local bodies. The next most endorsed measure was lifestyle modifications to minimize physical contact, avoidance of crowds, adopt non-touch greeting methods, adding nutritious food to boost immunity. All these measures are in line with the current health guidelines for the pandemic (49). Such accordance is suggesting that (inter)national guidelines have been effective in informing the opinion of the general public, and this is a very important observation, especially in the context of the sizeable misinformation surrounding COVID-19 and COVID-19 vaccines (50). About 20% of the votes supported only personal measures to quickly end the COVID-19 pandemic.

Regarding the collected ideas on what kind of measures can help to quickly end the COVID-19 pandemic, among the institution-mediated measures the most endorsed was healthcare accessibility and equity for general healthcare, vaccines, and tests (191 endorsements, Table 1). The world has witnessed disproportionately distributed healthcare facilities that negatively affected disadvantaged individuals, minority communities, and vulnerable populations around the world. The rapid spread of the pandemic indicated that the world was not ready with strategic planning to allocate limited resources to the highest possible benefit (51). The next most endorsed measure (189 endorsements) was the maintenance of a portfolio of vaccines and the adaptation of optimal immunization strategies. The need for the application of several alternative technologies for COVID-19 vaccine production was emphasized, along with some more specific approaches such as the passive vaccination of infants with breast milk or the introduction of plant-based vaccines (52). Whether vaccination alone would be enough to end the pandemic is still an open question (53). However, equitable distribution of vaccines to all corners of the world should be a priority, as whatever protection it provides should be shared with the global population. Interestingly, in the conducted Twitter poll (Figure 2) overall the importance of institutional measures alone received almost twice fewer votes (11%; a vote that was equal to the users who wanted to see the vote results only) in comparison to the significance of personal measures alone (20%), but in the ranking of the single recommended measures (Table 1), the highest-ranked personal measure ranked just third place (“Trust in experts and compliance with recommended measures;” 167 endorsements). Nevertheless, the poll results (Figure 2) unequivocally demonstrated that the majority (58%) of the users believed in the equal importance of efforts at the personal and institutional level to faster end the COVID-19 pandemic, whereby the superiority of the appropriate combination of approaches is also clearly in line with previous research findings (54).

## Conclusions

A popular social media platform, Twitter, was utilized successfully for crowdsourcing ideas on how to end the COVID-19 pandemic faster. During the 45 days of crowdsourcing, a total of seventeen suggested measures were yielded that received a total of 1,727 endorsements. Two distinct groups of measures have emerged from the ideas shared by Twitter users. One is personal measures, and the other group is institution-mediated measures. Personal endeavors include keeping trust in expert advice, lifestyle modifications, taking nutritional supplements, care for others, and psychological adjustments. Institutional measures that were suggested included accessibility and equality of healthcare access, generating a portfolio of vaccines, strategic funding, transparency in data presentation and raising scientific knowledge awareness, strengthening containment, free or affordable consumables like N95 masks, limiting commercialization of products needed to combat the pandemic, appropriate usage of disinfectants, rapid development of pharmaceuticals, punishments and rewards for denying and following recommended measures, and early detection and treatment of the disease. The measures framed by Twitter users were overall in line with many of the aspects of current national and international guidelines to combat the COVID-19 pandemic. In summary, our work exemplifies how Twitter can be utilized as the primary tool for crowdsourcing ideas with healthcare significance.

## Data availability statement

The original contributions presented in the study are included in the article/supplementary material, further inquiries can be directed to the corresponding authors.

## Author contributions

All authors listed have made a substantial, direct, and intellectual contribution to the work and approved it for publication.

## Funding

RS and BS acknowledge the support by the National Natural Science Foundation of China (32070671), the COVID-19 research projects of West China Hospital Sichuan University (Grant No. HX-2019-nCoV-057) as well as the Regional Innovation Cooperation between Sichuan and Guangxi Provinces (2020YFQ0019).

## Conflict of interest

The authors declare that the research was conducted in the absence of any commercial or financial relationships that could be construed as a potential conflict of interest.

## Publisher's note

All claims expressed in this article are solely those of the authors and do not necessarily represent those of their affiliated organizations, or those of the publisher, the editors and the reviewers. Any product that may be evaluated in this article, or claim that may be made by its manufacturer, is not guaranteed or endorsed by the publisher.

## Abbreviations

COVID-19: coronavirus disease 2019
DHPSP: Digital Health and Patient Safety Platform
mRNA: Messenger ribonucleic acid
FDA: Food and Drug Administration
EUA: Emergency Use Authorization
SARS-CoV-2: Severe acute respiratory syndrome coronavirus 2
PCR: Polymerase chain reaction.
